# Extracellular Vesicles: Potential Mediators of Psychosocial Stress Contribution to Osteoporosis?

**DOI:** 10.3390/ijms22115846

**Published:** 2021-05-29

**Authors:** Yangyang He, Karin Wuertz-Kozak, Linn K. Kuehl, Pia-Maria Wippert

**Affiliations:** 1 Medical Sociology and Psychobiology, University of Potsdam, 14469 Potsdam, Germany; kuehl@uni-potsdam.de (L.K.K.); wippert@uni-potsdam.de (P.-M.W.); 2 Faculty of Health Brandenburg, University of Potsdam, 14469 Potsdam, Germany; 3 Department of Biomedical Engineering, Rochester Institute of Technology (RIT), Rochester, NY 14623, USA; kwbme@rit.edu; 4 Schoen Clinic Munich Harlaching, Spine Center, Academic Teaching Hospital and Spine Research Institute of the Paracelsus Medical University Salzburg (AU), 81547 Munich, Germany; 5 Department of Psychology, Clinical Psychology and Psychotherapy, MSB Medical School Berlin, Rüdesheimer Straße 50, 14197 Berlin, Germany

**Keywords:** allostatic load, bone remodeling, microRNA, osteoblast, osteoclast

## Abstract

Osteoporosis is characterized by low bone mass and damage to the bone tissue’s microarchitecture, leading to increased fracture risk. Several studies have provided evidence for associations between psychosocial stress and osteoporosis through various pathways, including the hypothalamic-pituitary-adrenocortical axis, the sympathetic nervous system, and other endocrine factors. As psychosocial stress provokes oxidative cellular stress with consequences for mitochondrial function and cell signaling (e.g., gene expression, inflammation), it is of interest whether extracellular vesicles (EVs) may be a relevant biomarker in this context or act by transporting substances. EVs are intercellular communicators, transfer substances encapsulated in them, modify the phenotype and function of target cells, mediate cell-cell communication, and, therefore, have critical applications in disease progression and clinical diagnosis and therapy. This review summarizes the characteristics of EVs, their role in stress and osteoporosis, and their benefit as biological markers. We demonstrate that EVs are potential mediators of psychosocial stress and osteoporosis and may be beneficial in innovative research settings.

## 1. Introduction

Stress is a state in which homeostasis is threatened or perceived to be threatened [1]. In response to this threat, the activation of the hypothalamic-pituitary-adrenocortical axis will lead to increased secretion of glucocorticoids, which mobilizes energy to help the body respond to stressors, but chronic stress responses may be detrimental to the body’s health [2]. One consequence of the dysregulation of glucocorticoids is the increased glucose metabolism and the production of reactive oxygen species (ROS) within cells, leading to altered mitochondrial function and compromised integrity of mitochondrial DNA (mtDNA), systematic inflammatory processes, and accelerated cellular aging. The increase in glucose metabolism can also lead to the production of advanced glycation end products and subsequent acceleration of the cellular aging process through activation of its associated receptor [3,4]. Besides these singular physiological responses, there are long-term effects to be considered, better known as the Allostatic Load, defined as: “the wear and tear on the body,” and it refers to social, environmental, and psychological challenges, which accumulate as individuals are exposed to repeated or chronic stress [5].

Osteoporosis is an age-related bone disease characterized by reduced bone mass and bone microarchitecture destruction, resulting in decreased bone strength, increased bone fragility, and fracture risk [6]. Sustained stress can inhibit osteoblast activity and enhance osteoclast-mediated bone resorption, thus possibly leading to a decrease in bone mass in the long term [7]. However, cell-cell communications that exacerbate these processes are not well understood to date. In recent years, extracellular vesicles (EVs) have emerged as critical modulators of cell-cell communication in health and disease [8], and may be an essential player as mediators of stress-induced osteoporosis [9]. One of the psychological/physical stress response’s fundamental characteristics is sterile inflammation [10], i.e., inflammation that is not triggered by pathogenic bacteria, but by a physical, chemical, or metabolic harmful stimulation [11]. Danger/damage associated molecular patterns (DAMPs) play an essential role in psychological/physical stress-induced sterile inflammation. They are released from damaged or dying cells that activate the innate immune system by interacting with pattern recognition receptors [12]. Among the most relevant DAMPs are mtDNA, high-mobility group box 1, and S100 proteins, as well as heat-shock proteins [13,14,15,16]. The circulating EVs can maintain systemic immune homeostasis and regulate psychological stress-induced sterile inflammation by transmitting immunomodulatory signals [10]. Similarly, EVs can regulate the function of osteoblasts and osteoclasts, and consequently have a potential impact on osteoporosis [17]. Therefore, this review aims to evaluate whether EVs act as mediators of psychosocial stress and osteoporosis. To evaluate this research question, a thorough literature search was conducted using PubMed, Google Scholar, and Science Direct. Results are summarized here in the form of a narrative review.

## 2. The Characteristics of Extracellular Vesicles

EVs is a general term for numerous vesicles with a lipid bilayer membrane structure released by cells into the extracellular environment [18]. Based on their subcellular origin and biogenesis, EVs divide into three main categories: small EVs (also known as exosomes), medium/large EVs (also known as microvesicles), and apoptotic bodies [19]. Exosomes are vesicles with a ≈40–200 nm diameter and uniform size, which are released from intracellular multivesicular bodies (MVBs) fused with the cytoplasmic membrane [20,21,22]. In contrast, microvesicles are non-uniform particles ranging from 200–2000 nm in diameter that are formed and released from the cytoplasmic membrane in a budding manner. Apoptotic cells undergo programmed cell death and release apoptotic bodies (800–5000 nm in diameter), which share certain characteristics with microvesicles [23]. EVs carry multiple biomolecules, including DNA, RNA, proteins, glycans, lipids, and metabolites [24,25]. Thus, they can be used as cargoes to deliver information and alter the signaling pathways and biochemical composition of receptor cells. EVs can be derived from a variety of cells, such as mesenchymal stem cells (MSCs) [26], immune cells [27], tumor cells [28], platelets [29], and cardiomyocytes [30]. Furthermore, they can be detected in most body fluids, such as peripheral blood, breast milk, semen, urine, and saliva [31]. Thus, EVs have been recognized increasingly as promising biomarkers for the diagnosis and prognosis of several diseases.

**Figure 1:**
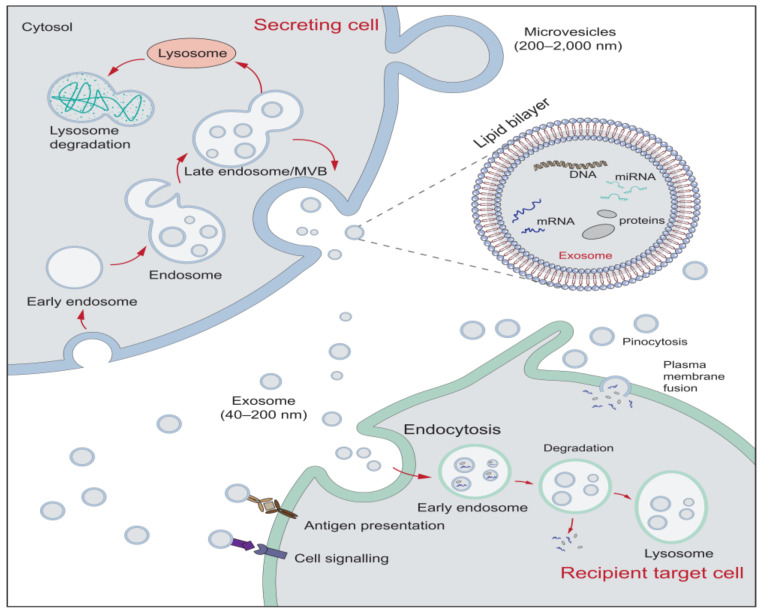
The biogenesis and secretion of EVs and their effects on target cells. The formation of exosomes begins with the endocytosis of the cell membrane. The endosome membrane sprouts inward to form vesicles, which transform into MVB. MVB can be sent to lysosomes for degradation or secreted into the exosomes (40–200 nm) by fusion with the plasma membrane. Microvesicles (200–2000 nm) are vesicles formed through a process of membrane budding or exocytosis. EVs can interact with target cells through receptor-mediated binding. Additionally, target cells can internalize EVs by target cells through endocytosis, pinocytosis, and plasma membrane fusion [9], where EVs can release their cargoes to affect target cells, or be degraded by lysosomes.

The composition of EVs has a crucial influence on their biological functions; as transmitters, EVs can activate cell surface receptor binding on target cells through proteins and bioactive lipid ligands, thereby inducing intracellular signaling and regulating the biological activity of the target cells. Besides, EVs can deliver their contents to target cells by fusing with the plasma membrane [17,32]. Figure 1 shows the biogenesis and secretion of EVs and their effects on target cells. Studies on EVs show that they have a complex composition, including lipids, proteins, nucleic acids, and other metabolites. These components play an essential role in the function of EVs. Nucleic acids carried by EVs can be potential biomarkers because of their genetic characteristics [33]. Current research is more focused on microRNA (miRNA, miR). MiRNAs are 17–24 nucleotide endogenous, non-coding RNAs, which post-transcriptionally silence target genes’ expression by binding to the 3’-untranslated region (UTR) open reading frame region of target messenger RNAs [34,35], thus playing a vital regulatory role in the organism. Because of the potential relevance of miRNAs as disease markers and therapeutic tools, it is of great importance to further our understanding of their biological properties and functions [36,37]. The roles of EVs in human tissues are listed in Table 1.

## 3. The Role of EVs in the Stress Response

### 3.1. EVs May Serve as Biomarkers for Psychosocial Stress

Both psychosocial and metabolic stresses may act through common underlying subcellular mechanisms, with mitochondria as critical players [51]. Psychosocial stress disrupts adaptive glucocorticoid signaling and glucose levels, which alters mitochondrial structure and function, increasing ROS production within cells, producing oxidative stress and cellular damage, and promoting systemic inflammation [52]. Moreover, ROS can regulate miRNA expression through epigenetic modification and transcription factors [53]. EVs can carry multiple miRNAs involved in intercellular communication; it was previously shown that various miRNAs associated with inflammation and oxidative stress are increased in plasma EVs isolated from human immunodeficiency virus-positive subjects on antiretroviral therapy and may, thus, function as biomarkers of targetable pathways leading to disease pathogenesis [54]. Hence, the changed miRNAs in EVs may serve as potential biomarkers for the psychosocial stress process. Psychosocial stress induces neuroendocrine mediators that cause a structural and functional realignment of mitochondria, constituting mitochondrial allostatic load [55]. As an extension of the allostatic load model, the prolonged activation of allostatic mechanisms at the mitochondrial level (excessive mitochondrial fragmentation, ROS production leading to mtDNA damage and respiratory insufficiency, and release of pro-inflammatory molecules) constitutes the mitochondrial allostatic load [52]. Changes in mtDNA levels have been reported in many human diseases, such as Parkinson’s disease, acute kidney injury, and cancer [56,57,58]. Thus, researchers proposed that mtDNA levels in body fluids and tissues may be a biomarker of mitochondrial dysfunction. Since mtDNA is present in EVs and can act on target cells through EVs’ transport [59,60], the same as miRNA, the changed mtDNA in EVs may also act as potential biomarkers for the psychosocial stress process. In the stress response, mitochondria also cooperate with the endoplasmic reticulum [61]; therefore, the endoplasmic reticulum may be involved in the stress response process. Research has shown that a combination of physical/psychological and biological stress enhances endoplasmic reticulum stress [62]. Severe endoplasmic reticulum stress-mediated release of EV-associated DAMPs may be associated with specific chronic inflammatory diseases [16]. Thus, the role of endoplasmic reticulum in psychosocial stress is worth studying in the future.

### 3.2. Stress Modifies miRNAs in EVs to Regulate the Immune Response

Psychosocial stress may regulate immune functions, and the role of EVs in the immune response has also been highlighted [63]. We focus on the potential role of circulating EVs as a transmitter of immune-regulatory signals. Multiple stress models have demonstrated that miRNAs in EVs may be involved in the regulation of immunity. One recent study showed that the exposure of rats to an acute stressor (inescapable tail shock) resulted in altered miRNA expression in circulating plasma exosomes (decreased miR-142-5p and miR-203) [64]. These altered miRNAs in exosomes are likely to be an essential component of stress-induced immune regulation. Previous research has shown that reduced expression of miR-142-5p increases T cell function and promotes B cell hypersensitivity [65]. Furthermore, miR-203 can target the suppressor of cytokine signaling-3 (SOCS-3), a negative regulator of IL-6 and interferon-γ induced signaling pathways [66,67]; SOCS-3 also affects inflammatory responses by inhibiting IL-2 and IL-12 signaling [68,69]. The suppression of SOCS-3 by miR-203 may lead to an increased inflammatory response. In summary, stress leads to the inhibition of miR-203 expression, which results in the activation of SOCS-3 and its inhibition of pro-inflammatory cytokines.

In addition to acute stress, chronic unpredictable mild stress (CUMS), a model of depression, can affect miRNA expression contained in serum EVs in rats (23 upregulated and 34 downregulated), with possible immunological consequences [70]. MiR-128-3p, which is upregulated after CUMS, stimulates gene expression of pro-inflammatory cytokines (Ccl5, Cx3cl1, and Cxcl7). Moreover, Shyamasundar et al. [71] show that miR-128-3p regulates inflammation in the normal rat kidney. The overexpression of miR-26a-5p (also upregulated after CUMS) attenuated the inflammatory response in mice with lipopolysaccharide-induced acute lung injury by decreasing total protein, neutrophil, and lymphocyte counts and expression levels of TNF-α, IL-1β, and IL-6 in bronchoalveolar lavage fluid [72]. Moreover, miR-455-5p, which was downregulated after CUMS, could specifically bind to SOCS-3 3’-UTR and inhibit SOCS-3 expression [73], thereby participating in the inflammatory response. The effects of specific stressors on miRNAs in EVs are listed in Table 2.

## 4. The Role of EVs in Osteoporosis

### 4.1. Overview of Osteoporosis and Bone Remodeling

As one of the human body’s essential tissues, bone needs sufficient stiffness and toughness to maintain bone strength to avoid fractures. In terms of the body’s natural processes, the positive balance between bone formation (by osteoblasts) and bone resorption (by osteoclasts) before adulthood increases bone mass and reaches its peak (typically achieved at different skeletal sites from 25 to 35 age years [76]), and bone remodeling balance maintains bone mass in adulthood. However, with increasing age, most bone loss occurs during and after menopause.

Bone remodeling, a lifelong process, refers to bone formation (form new bone tissue) and bone resorption (remove mature bone from the skeleton). This process involves skeletal-related cells, such as osteoclasts, osteoblasts, osteocytes, and several immune cells, such as T cells, B cells, and megakaryocytes [77]. Bone remodeling occurs in the basic multicellular unit, consisting of osteoblasts, osteoclasts, and osteocytes within the bone-remodeling cavities [78]. The process begins with bone-resorbing osteoclasts, followed by bone-forming osteoblasts, and in normal bone, the remodeling cycle results in complete filling of the resorption cavity with new bone [78,79]. Osteocytes, the most abundant cells in bone tissues, can sense and respond to environmental mechanical stimuli and regulate bone formation and bone resorption [80]. Thus, osteocytes are the central coordinator of bone reconstruction and mineral homeostasis. In the bone remodeling process, runt-related transcription factor 2 (Runx2) and Osterix plays an essential role for osteoblast differentiation [81,82], and the osteoclast differentiation is mainly regulated by the receptor activator of nuclear factor κ-Β ligand (RANKL)/receptor activator of nuclear factor κ-Β(RANK)/osteoprotegerin pathway. Namely, osteoblasts can produce RANKL, which can bind to RANK on osteoclasts’ precursor, thus promoting osteoclast differentiation. To tightly regulate osteoclastogenesis, osteoblasts also secrete osteoprotegerin to compete with RANK to bind RANKL, thus inhibiting osteoclast differentiation [83].

### 4.2. EVs Regulate Osteoclasts Differentiation and Activity

MiRNAs, as one of the cargoes carried by EVs, have a vital role in bone homeostasis. For example, the highly expressed miR-503-3p in EVs released by osteoblasts can inhibit osteoclastogenesis by inactivating the RANK/RANKL signaling pathway [84,85]. Besides, blood vessels play an essential role in bone repair and regeneration [86]. A study by Song et al. [87] demonstrated that EVs derived from the vascular endothelial cell have more effective bone targeting than those derived from osteoblast or bone marrow mesenchymal stem cells (BMSCs) and can inhibit the activity and differentiation of osteoclasts through miR-155. Thus, the miR-155-containing EVs may be a potential target against osteoporosis. Interestingly, some tumor cells can affect osteoclast function by secreting EVs. Increased expression of miR-21 was observed in EVs derived from lung adenocarcinoma cells, which promoted osteoclastogenesis by targeting programmed cell death protein 4 [88]. Similarly, breast cancer cells secrete miR-20a-5p-containing EVs, which promote the proliferation and differentiation of osteoclasts [89].

EVs can affect bone remodeling by directly regulating osteoclast differentiation and activity. Huynh et al. [90] found that the EVs derived from osteoclast precursors stimulate the formation of vitamin D-dependent osteoclasts. However, EVs from osteoclast-enriched cultures inhibited osteoclastogenesis. The results of this experimental study show that the EVs from mature osteoclasts contain RANK, which could competitively inhibit the stimulation of RANK on the osteoclast surface, similar to the role of osteoprotegerin mentioned above. Besides, the RANK-containing EVs can use the RANK/RANKL interaction to target RANKL-expressing cells to transfer regulatory molecules [90]. Moreover, osteoblasts can affect osteoclasts by secreting EVs. The RANKL-containing EVs released by osteoblasts are transferred to the precursors of osteoclasts, thus stimulating RANKL/RANK signal transduction and promoting the formation of osteoclasts [91]. To better understand the role of EVs in osteoblast-osteoclast communication, researchers loaded osteoblast-derived EVs with osteoclast-inhibiting drugs (zoledronate and dasatinib). They found that osteoblast EVs internalized and shuttled osteoclast-inhibiting drugs to inhibit osteoclasts’ activity in vivo and in vitro [92], which opens up an avenue for the use of EVs in the treatment of bone diseases. The above studies show that EVs from a variety of cells can regulate osteoclasts.

### 4.3. EVs Affect Osteoblasts and Osteogenic Function

Osteoblasts are the bone-forming cells of remodeling units and are crucial for skeletal growth and maintenance [93]. As mentioned above, osteoblasts can secrete EVs to influence osteoclast function. In turn, osteoclasts can secrete EVs that modulate osteoblast activity. Sun et al. [94] found that osteoclasts secrete miR-214-containing EVs, specifically recognizing osteoblasts through the ephrina2/ephrin type-A receptor 2 interaction. Moreover, miR-214 directly targets activating transcription factor 4 to inhibit bone formation [95]. The osteoclast-derived EVs exist not only in the bone microenvironment but they can also enter the blood. Researchers found upregulated levels of miR-214 in serum EVs of osteoporotic patients, which means that miR-214 in EVs serve as a potential biomarker of bone loss [94]. Likewise, osteoclasts-derived miR-23a-5p-containing EVs inhibit the activity of osteoblasts by targeting Runx2 [96]. Therefore, the EV-mediated intercellular communication between osteoblasts and osteoclasts may be a new direction for the study of bone remodeling mechanisms.

MSCs are known to stimulate tissue regeneration. Furthermore, EVs released from MSCs have attracted much attention in bone research. A recent study showed that BMSCs-derived EVs could regulate osteoblast differentiation and osteogenic gene expression in vitro, thus improving osteogenic function [97]. Additionally, MSCs-derived EVs induce osteogenic differentiation and mineralization during the late stages of osteogenic differentiation. Furthermore, target prediction of differentially expressed miRNAs in EVs suggests a significant enrichment of signaling pathways regulating osteogenic differentiation [98]. Some researchers have explored the possible clinical applications of BMSCs based on previous literature. For example, Fang et al. [99] found that BMSCs-derived EVs significantly reverse the decreased osteogenic differentiation of BMSCs in steroid-induced femoral head necrosis, thus serving as a potential therapeutic strategy for steroid-induced femoral head necrosis. These studies reveal the potential application of MSCs-derived EVs in bone regeneration therapy. Many studies support the role of EVs in bone remodeling, shown in Table 3, but it is not discussed in detail.

## 5. EVs as Potential Mediators of Psychosocial Stress and Osteoporosis

Considering that EVs play an essential role in intercellular communication, are involved in psychosocial stress, and affect osteoporosis progression, one can hypothesize that EVs may constitute a molecular link between psychosocial stress and osteoporosis. As mentioned above, previous literature demonstrated the effects of multiple stress models on miRNAs in EVs and the role of altered miRNAs in EVs during the progression of osteoporosis. Regarding the effects of acute stress on EVs, Beninson et al. [64] showed that the exposure of rats to acute stressors resulted in decreased miR-142-5p expression in plasma exosomes. Since miR-142-5p can stimulate osteoblast activity and matrix mineralization [105], the miR-142-5p-containing exosomes may mediate between stress and osteoporosis. However, as the formation of osteoporosis is a chronic process, the focus was on the description of the possible effect of EVs’ changes on bone homeostasis under chronic psychosocial stress.

Regarding the effects of the depression model on EVs, Fang et al. [70] reported that chronic unpredictable mild stressors, which can lead to allostatic overload, induce changes in miRNA content in serum EVs of rats. The most significantly upregulated miRNAs expression in serum EVs in rats exposed to chronic unpredictable mild stressors (miR-126a-3p and miR-128-3p) plays a role in bone remodeling. MiR-126a-3p inhibits the osteogenesis of human adipose-derived mesenchymal stem cells by blocking Wingless and Int-1 (Wnt) activation [106] because Wnt signaling cascade leads to bone formation and the inhibition of bone resorption [107]. Besides, miR-128-3p can inhibit the osteogenic ability of MSCs [108]. On the other hand, the most significantly downregulated miRNAs expression in serum EVs in chronic unpredictable mild stressors stimulated rats (miR-455-3p and miR-187-5p) also exhibited an association with bone remodeling. MiR-455-3p has the effect of protecting osteoblasts from oxidative stress, which is a risk factor for osteoporosis, thus promoting osteoblasts growth [109]. Additionally, miR-187-5p promotes differentiation of BMSCs to osteoblasts [110]. However, chronic unpredictable mild stressors lead to a downregulated miR-23a-3p expression in EVs [70]. In turn, the inhibition of miR-23a-3p promotes osteoblast proliferation and differentiation [111], which contradicts the above findings. Nevertheless, several miRNAs with the most significant alterations in mice’s serum EVs after stimulation with chronic unpredictable mild stressors were shown to harm bone formation and may be involved in osteoporosis progression. The currently known EVs associated with stress and bone are listed in Table 4.

**Figure 2:**
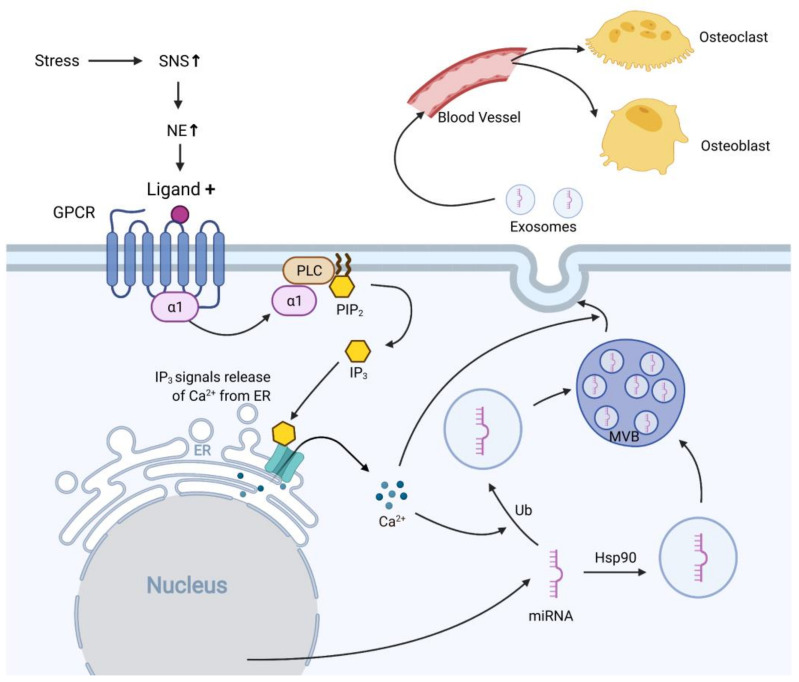
The potential mechanisms of EV involvement in psychosocial stress contributed osteoporosis. Psychosocial stress induces the release of norepinephrine (NE) from sympathetic nerve terminals by activating the sympathetic nervous system (SNS); the released norepinephrine can bind to the α1-adrenergic receptor, which is coupled with the G-protein coupled receptor (GPCR). GPCR dissociates upon receptor activation and promotes phospholipase C (PLC), catalyzing the breakdown of phosphatidylinositol bisphosphate (PIP_2_) into inositol trisphosphate (IP_3_). IP_3_ binds to the IP_3_ receptor on the endoplasmic reticulum (ER), leading to elevated cytosolic Calcium (Ca^2+^) [117]. Cytosolic Ca^2+^ increases ubiquitination (Ub) and targets specific miRNAs to endosomes, and other miRNAs target endosomes via heat shock protein 90 (HSP90) [118]. Then, the endosomes are directed to the multivesicular bodies (MVBs). Through the mediation by Ca^2+^, the MVBs fuse with the cell’s plasma membrane, releasing endosomes into the extracellular space, where they are considered exosomes [64,119]. The circulating exosomes are internalized by osteoblasts or osteoclasts, where they release the genetic materials they carry, impact their physiological function, and thus, participate in osteoporosis development.

A leading question is whether the chronic psychosocial stress-modified circulating EVs can target bone tissue and affect its function. Even though studies that directly investigate this question are missing so far, some studies linked osteocyte EVs and circulating EVs. One study showed that osteoblasts released EVs containing specific miRNAs circulating in the bloodstream and transferred their contained biological components to receptor cells [112]. On the contrary, distal tissues can also affect bone tissue by secreting EVs. For example, EVs derived from growth hormone-secreting pituitary adenoma can be internalized by osteoblasts, promoting osteoblast proliferation and bone formation [113]. These studies have demonstrated that EVs can act as mediators to participate in the biological effects of bone and other tissues. Although no direct proof that chronic psychosocial stress-modified circulating EVs can target bone, previous literature on the role of EVs in intercellular communication shows that EVs are a promising candidate as a mediator of chronic psychosocial stress-related effects on bone. The potential mechanisms of EV involvement in chronic stress-induced osteoporosis are shown in Figure 2.

## 6. Conclusions and Perspective

Since the discovery of EVs, their intrinsic properties have attracted much attention. The intercellular communication mechanism by EVs is probably indispensable for systemic communication. However, it remains uncertain whether EV-mediated transport of biological cargoes can alter target cell function in a real physiological setting. Unlike laboratory experiments, in which large amounts of purified EVs were added to cells, the situation in real physiological settings is often more complex and variable. Moreover, the delivery efficiency of EVs is not yet fully elucidated. Therefore, further studies are needed to determine the function of EVs and their possible clinical applications (as biomarkers and therapeutics) in real-life settings.

In conclusion, this review summarizes the effects of stress on EVs and their role in osteoporosis development. Many studies demonstrated that psychosocial stress is a risk factor for osteoporosis. However, no studies have taken the perspective of EVs as a mediator of the association between psychosocial stress and osteoporosis. As shown in previous research, many miRNAs in EVs affected by stress also impact osteoporosis progression. This underlines the possibility that miRNAs in EVs may constitute a molecular link between stress and osteoporosis. However, whether EV-mediated miRNA alterations can modulate the interaction between psychosocial stress and bone metabolism in a real physiological setting remains unclear. Additionally, the specific molecular mechanisms of their action will require further investigation. Future studies should identify psychosocial stress-modified circulating (plasma or serum) EVs to understand their role in osteoporosis, which could change the current perspective on how psychosocial stress contributes to osteoporosis. Since current studies on the mechanisms of psychosocial stress contributed osteoporosis are mainly about the sympathetic nervous system or hypothalamic-pituitary-adrenocortical axis, we recommend exploring the possible role of EVs in psychosocial stress-mediated development of osteoporosis, thereby possibly paving the way towards novel diagnostic and therapeutic tools.

## Figures and Tables

**Table 1:** Role of EVs in human tissues.

Tissue	Functions	Reference
Tumor	Biomarker Alters tumor microenvironment Regulates tumor immune response Involved in tumor angiogenesis	[31,32,38,39]
Bone	Biomarker Regulates osteogenic differentiation of mesenchymal stem cells Regulates osteoblast proliferation and activity Affects osteoblast differentiation Regulates osteoclast function and induces osteoclast differentiation	[17,40,41,42]
Heart	Biomarker Promotes angiogenesis Cardioprotection and regeneration	[43,44]
Brain	Biomarker Influences inflammatory and regulatory pathways in the brain Neuroprotective effect	[45,46,47]
Kidney	Biomarker Involved in the development of renal fibrosis Contributing to kidney repair	[48]
Gastro-intestinal tract	Immunomodulation Response of anti-apoptotic, antioxidant stress Regulates the homeostasis of gut microbiota	[49,50]

**Table 2:** The effects of specific stressors on miRNAs in EVs.

Stressors	MiRNAs in EVs	Source of EVs	Stress-Induced Change	References
Chronic unpredictable mild stress	miR-139-5p	Blood and brain from mice	↑	[74]
Chronic unpredictable mild stress	miR-126a-3p, miR-128-3p, miR-26a-5p, miR-191a-5p	Serum from rats	↑	[70]
Mechanical stress	miR-1246	Fibroblast	↑	[75]
Mechanical stress	miR-133a-3p, miR-203-3p	Fibroblast	↓	[75]
Chronic unpredictable mild stress	miR-455-3p, miR-187-5p, miR-206-3p, miR-455-5p	Serum from rats	↓	[70]
Inescapable tail shock	miR-142-5p, miR-203	Plasma from rats	↓	[64]

“↑” means upregulated; “↓” means downregulated.

**Table 3:** A summary of EVs associated with bone remodeling.

Source	Bioactive Factors Containing	Target	Function	References
Osteoclasts	RANK	Osteoclasts	Inhibits osteoclast formation	[90]
Osteoclasts	miR-214	Osteoblasts	Inhibits the activity of osteoblasts through ephrina2/ephrin type-A receptor 2 interaction and targets activating transcription factor 4 to inhibit bone formation	[94,95]
Osteoclasts	miR-23a-5p	Osteoblasts	Inhibits the activity of osteoblasts by targeting Runx2	[96]
Osteoclasts	miR-214-3p	Osteoblasts	Inhibits osteoblastic bone formation	[100]
Osteoblasts	RANKL	Osteoclast precursors	Facilitates osteoclast formation by binding RANK on the osteoclast precursor surface	[91]
Osteoblasts	RANKL	Osteoclasts	Induces the apoptosis of osteoclasts	[92]
Preosteoblasts	TRIP-1	The extracellular matrix of bone	Promotes mineralization	[101]
BMSCs	miR-196a	Osteoblasts	Improves osteogenic function	[97]
BMSCs	miR-885-5p	BMSCs	Inhibits osteogenic differentiation by repressing Runx2	[102]
BMSCs	miR-151-5p	BMSCs	Promotes osteogenic differentiation	[103]
Endothelial cells	miR-155	Osteoclasts	Inhibits the activity and differentiation of osteoclasts	[87]
Endothelial cells	miR-31	MSCs	Inhibits osteogenic differentiation by repressing Frizzled-3	[104]

BMSCs: Bone marrow mesenchymal stem cells; MSCs: Mesenchymal stem cells; RANK: Receptor activator of nuclear factor κ-B; RANKL: Receptor activator of nuclear factor κ-Β ligand; TRIP-1: Transforming growth factor beta receptor II interacting protein-1; Runx2: Runt-related transcription factor 2.

**Table 4:** The currently known EVs associated with psychosocial stress and bone.

MiRNAs in EVs	Stress-Induced Change	The Effect of MiRNAs in EVs on Bone	References
miR-126a-3p	↑	Inhibits the osteogenesis of human adipose-derived mesenchymal stem cells	[70,106]
miR-128-3p	↑	Inhibits the osteogenic differentiation of MSCs	[70,108]
miR-26a-5p	↑	Inhibits the osteogenic differentiation of mouse adipose-derived mesenchymal stem cells	[70,114]
miR-139-5p	↑	Inhibits BMSC osteogenesis by targeting Wnt/β-catenin signaling pathway	[74,115]
miR-455-3p	↓	Protection of osteoblasts from oxidative stress	[70,109]
miR-187-5p	↓	Promotes differentiation of BMSCs to osteoblasts	[70,110]
miR-1-3p	↓	Stimulates the osteogenesis of mouse MSCs and inhibits their adipogenesis	[70,116]
miR-23a-3p	↓	Inhibits the osteogenesis	[70,111]

“↑” means upregulated; “↓” means downregulated; BMSCs: Bone marrow mesenchymal stem cells; MSCs: Mesenchymal stem cells; Wnt: Wingless and Int-1.

## Abbreviations

BMSC	Bone marrow mesenchymal stem cell
CUMS	Chronic unpredictable mild stress
DAMPs	Danger-associated molecular patterns
ER	Endoplasmic reticulum
EVs	Extracellular vesicles
GPCR	G-protein coupled receptor
HSP	heat-shock protein
IP_3_	Inositol trisphosphate
miRNA, miR	microRNA
MSCs	Mesenchymal stem cells
mtDNA	mitochondrial DNA
MVBs	Multivesicular bodies
PIP_2_	Phosphatidylinositol bisphosphate
PLC	Phospholipase c
RANK	Receptor activator of nuclear factor κ-Β
RANKL	Receptor activator of nuclear factor κ-Β ligand
ROS	Reactive oxygen species
Runx2	Runt-related transcription factor 2
SNS	Sympathetic nervous system
SOCS-3	Suppressor of cytokine signaling-3
Ub	Ubiquitination
UTR	Untranslated region
Wnt	Wingless and Int-1
